# The Impact of Tamoxifen Usage in Breast Cancer Patients on the Development of Histopathological Lesions in the Cervix Uteri

**DOI:** 10.3390/medicina60081268

**Published:** 2024-08-06

**Authors:** Ferhat Cetin, İlkan Kayar, Goksu Goc, Özer Birge

**Affiliations:** 1 Department of Obstetrics and Gynecology, Private Osmaniye Park Hospital, 80010 Osmaniye, Turkey; 2 Selimiye, Bölge Trafik Yanı, Musa Şahin Bulvarı, Merkez, 80010 Osmaniye, Turkey; 3 Department of Obstetrics and Gynecology, Osmaniye State Hospital, 80000 Osmaniye, Turkey; ilkankayar@gmail.com; 4 Department of Obstetrics and Gynecology, American Hospital, 10000 Prishtine, Kosovo; goksu.goc@gmail.com; 5 Department of Gynecological Oncology, Niger Turkey Friendship Hospital, Niamey 920271, Niger; ozbirge@gmail.com

**Keywords:** cervix uteri, histopathology, human papillomavirus, breast cancer, tamoxifen

## Abstract

*Background and Objectives*: The purpose of the present study was to compare the results of colposcopic biopsies in patients with breast cancer and those who tested positive for HPV in cervix uteri cytological screenings, with a control group of HPV-positive individuals without breast cancer. Additionally, through this study, we aimed to investigate the impact of tamoxifen treatment, an anti-oestrogen drug used following breast cancer treatment, on histopathological changes. Breast cancer is the most common type of cancer and cause of death in women worldwide. Cervical cancer ranks as the second most prevalent form of cancer among women globally, with prevalence rates ranking just behind those of breast cancer. Human papillomavirus (HPV) positivity is a requirement for the development of cervical cancer, although it is not the sole factor responsible. *Materials and Methods*: A comparison was made between the histopathological results of 52 patients diagnosed with breast cancer, who tested positive for HPV in routine cervical cytological screenings and underwent colposcopic biopsy, and 230 cases without any abnormalities. A study was conducted to compare healthy individuals between the ages of 30 and 65 who were diagnosed with breast cancer and those who did not have breast cancer. The participants underwent HPV screening as part of the national cervical cytology screening programme. *Results*: The average age of those diagnosed with breast cancer was 46.73 ± 7.54; in comparison, the average age of participants in the control group was 47.49 ± 7.95. There was no statistically significant difference in age between the two groups (*p*: 0.530). A total of 51 cases (98.1%) of breast cancer were found to have actively used the anti-oestrogen drug tamoxifen for a duration ranging from at least 6 months to 5 years. One patient (1.9%) in the breast cancer group did not use tamoxifen. During routine cervical cytological screenings, it was observed that both breast cancer cases and healthy cases tested positive for HPV. The most commonly detected types of HPV in both groups were HPV 16 and 18, with rates of 73.1% noted in the breast cancer group and 92.6% noted in the healthy group, results consistent with the rates found in the general population. HPV 16 was found in 58.7% of participants in the control group and 42.3% of participants in the breast cancer group. There was a statistically significant difference between the two groups (*p*: 0.032). There was no statistically significant difference observed between the two groups in terms of normal, high-grade cervical intraepithelial lesions (HGSILs); low-grade cervical intraepithelial lesions (LGSILs); and chronic cervicitis histopathological lesions based on colposcopic and endocervical biopsy results, smear cytology, and HPV results (*p*-values of 0.913 and 0.877, respectively). *Conclusions:* Our study results indicate that tamoxifen treatment, an anti-oestrogen drug administered for chemoprevention purposes in the management of breast cancer, does not lead to an increase in abnormal histological changes in the cervix uteri. In all cases of breast cancer, gynaecological examination and cervical cytological screening should be advised.

## 1. Introduction

Breast cancer is the most common type of cancer and cause of death in women worldwide [1,2]. The worldwide rate of breast cancer stands at 38.9 per 100,000 individuals [3].

Breast cancer appears to metastasise to all areas of the body, such as the lymph nodes, the liver, bone tissue, the lungs, and the brain. It should also be noted that this type of cancer has the potential to metastasise in lower genital organs, such as the cervix uteri, with this form of metastasis being very rare [4].

Risk factors for breast cancer include age, mutations in the BRCA (Breast Cancer) 1 and BRCA (Breast Cancer) 2 genes, a family history of breast cancer in two or more first-degree relatives at an early age, a past history of cancer in one breast, high breast density in mammography in postmenopausal women, breast cancer in a first-degree relative, high levels of radiation exposure, being in a postmenopausal state, the first pregnancy being beyond the age of 35, experiencing menarche before the age of 12, going through menopause after the age of 55, being obese after menopause, and using hormone replacement therapy for a lengthy period of time [5]. Treatment modalities for breast cancer involve the use of surgery, chemotherapy, and radiation, either individually or in conjunction. The course of treatment includes adjuvant chemotherapy and/or radiation and smart medication therapies. Subsequently, anti-oestrogen therapy involves the use of selective oestrogen receptor agonists and aromatase inhibitor treatments [3,5].

Cervical cancer ranks as the second most prevalent cancer among women globally, with prevalence rates ranking just behind those of breast cancer [6,7]. A globally recognised screening test for cervical cancer that adheres to international criteria has been developed. There has been a decline in the incidence of cervical cancer and the mortality rates of patients with this disease in the last five decades with the introduction of cervical cancer screening programmes in several developed countries. Cervical neoplasia is caused by the persistent infection of one or more oncogenic strains of human papillomavirus (HPV) [8]. Cervical cancer is an uncommon long-term outcome of disseminated viral infection affecting the cervical epithelium.

Human papillomavirus (HPV) positivity is a requirement for the development of cervical cancer, although it is not the sole factor responsible. Based on studies and physio-pathological evaluations, it is emphasised that the presence of HPV is necessary for the development of cervical cancer. Other risk factors either enhance the likelihood of being exposed to the virus or play a significant role in accelerating the process of cancer development through viral persistence. Roughly 70% of sexually active adults are exposed to human papillomavirus (HPV) at some stage in their lifetime. Additionally, it has been noted that over 70% of such individuals fall between 15 and 24 years of age [9,10].

In recent years, it has been stated in the literature that the use of HPV DNA chip tests coupled with the Pap smear test is advantageous in increasing the accuracy of screening tests in the early diagnosis of cervical cancer, especially in order to increase the sensitivity of cervix uteri cytological tests [11].

Cervical cancer screening programmes play a crucial role in reducing the occurrence and mortality rates associated with this type of cancer. Our country (Turkey) is now making efforts to establish cervical cancer screening programmes that utilise a number of different technologies, including Pap (Papanicolaou) smear and HPV DNA screening. The goal is to extend these screening programmes to the entire population [10]. The process of examining a cervical smear for cellular abnormalities and performing simultaneous HPV DNA testing on the same sample is referred to as a “co-test”. At present, co-testing is widely recognised as the preferred screening method for women aged 30 years or older.

Tamoxifen is a tolerable, selective oestrogen receptor modulator with limited side effects that provides chemoprevention through its anti-estrogenic effect in cases of oestrogen-receptor-positive breast cancer with a non-steroidal partial agonistic effect. The results of a number of studies have demonstrated that the above substance has anti-estrogenic properties in breast cancer tissue that expresses oestrogen receptors. However, in the female genital system, it has a proliferative impact on the endometrium, cervix, and vaginal epithelium. This effect is mediated by its ability to activate oestrogen receptors [12,13]. Therefore, particularly in breast cancer cases, it is imperative to perform thorough examinations of the cervix, vagina, and most importantly, the endometrium epithelium for any pathophysiological alterations when tamoxifen is administered [14]. It has also been stated that cervical cytological Pap smear tests are particularly beneficial for the early detection of cervical uteri, vaginal, and endometrial epithelial alterations [15].

The results of previous studies indicate that tamoxifen stimulates the proliferation of the cervicovaginal epithelium through oestrogen receptors, specifically in relation to cervix uteri lesions identified using standard co-tests or cytological screening tests conducted worldwide [16,17,18].

While several studies have examined the impact of tamoxifen on endometrial alterations in breast cancer patients, there is a scarcity of research on its effects on the cervix, vagina, and vulva within the female genital system. The presence of oestrogen receptors in both the squamous and glandular cells of the cervix uteri has been noted in the scientific literature. These receptors are particularly prominent during the pre- and post-menopausal stages of a woman’s life. It is believed that the number and affinity of these receptors remain constant throughout the menstrual cycle. Furthermore, tamoxifen is believed to exert its effects on the cervicovaginal tissues by interacting with these receptors [19,20].

Chemotherapy, radiotherapy, smart medication treatments, and anti-oestrogen treatments are commonly used as adjuvant therapies following surgery, which is the major treatment approach utilised for breast cancer. Such treatments can lead to pathophysiological alterations in all cells of the body, particularly when the immune system is suppressed. It is believed that the cells in the cervical uteri of women of reproductive age, particularly during the phase of rapid growth and shedding, may experience rapid alterations following immune system suppression caused by post-cancer treatments. This transformation may be pathologically expedited in cases where HPV DNA positivity is present [21].

The aim of the present study is to discuss the effect of tamoxifen use in breast cancer cases on cervix uteri histopathological lesions in HPV-positive cases with the help of the presently available literature on the subject.

## 2. Materials and Methods

A retrospective analysis was conducted on the medical records of 52 patients diagnosed with breast cancer and 230 healthy patients without breast cancer who visited our clinic for gynaecological complaints between January 2019 and 2024 in Osmaniye State Hospital in Turkey. All of the patients analysed were diagnosed as being HPV positive after undergoing clinical examination and radiological and laboratory evaluations. The analysis was performed using the electronic archive system, following the approval of the ethics committee on 27 December 2023 (ethics committee reference number: E-773-78720-774.99-240236181).

We aimed to exclude certain cases from the analysis, specifically those that were HPV negative; had recurrent breast cancer; were suffering from a second primary or metastatic cancer; were pregnant; were under 30 years of age or over 65 years of age; had used or were currently using aromatase inhibitors; had received the HPV vaccine; were HIV positive; were HSV positive; were CMV positive; were Toxoplasma positive; had chronic immune system disorders; or had previously undergone conisation, cryotherapy, cauterisation, or similar cervical uteri operations. The present study included individuals between the ages of 30 and 65 who were diagnosed with breast cancer and underwent surgery, underwent chemotherapy, and/or used tamoxifen as an anti-oestrogen drug for at least 6 months to 5 years. These individuals had no recurrence of breast cancer. The control group consisted of individuals who had cytological abnormalities and tested positive for HPV after a normal cervical cytological screening.

The histopathological results of 52 women diagnosed with breast cancer who were found to be HPV positive in routine cervical cytological screening and underwent colposcopic biopsy were compared with the histopathological results of 230 HPV-positive cases in which no abnormalities were detected according to the Bethesda 2001 classification [8].

Individuals between 30 and 65 years of age who were diagnosed with breast cancer and healthy individuals who did not have breast cancer underwent HPV screening as part of the national cervical cytology screening programme and were evaluated in terms of age, gravida, parity, co-morbid diseases, contraceptive methods, menopausal status, anti-oestrogen medication usage, steroid usage, insulin usage, smoking, positive HPV types, and histopathological results following colposcopy.

The HPV-positive controls and breast cancer cases were subjected to colposcopic examination. Biopsies taken as a result of colposcopic examination were evaluated histopathologically according to the Bethesda 2001 classification [8].

## 3. Statistical Analyses

The statistical analysis of the study results was conducted using the IBM SPSS Statistics 22.0 programme. During the evaluation of the study data, descriptive statistical methods such as the mean and standard deviation were used. Additionally, Student’s *t*-test was employed to compare parameters with a normal distribution between two groups in the analysis of quantitative data. Conversely, the Mann–Whitney U test was utilised to compare parameters that did not exhibit a normal distribution between the two groups. The chi-squared test, Fisher’s exact test, and the continuity correction (Yates) test were employed to compare the qualitative data. Significance was assessed at a level of *p* < 0.05.

## 4. Results

The present study was carried out from January 2019 to 2024, including a sample size of 282 cases. Among the cases, 52 patients were diagnosed with breast cancer, and the remaining 230 cases constituted the control group. The patients’ ages covered a range of 31 to 65 years, with an average age of 47.35 ± 7.86 years. There was no statistically significant difference between the average ages of participants in the different groups (*p* > 0.05).

Participants in the different groups did not exhibit a statistically significant difference in terms of gravida, parity, abortion, and dilation/curettage (D/C) numbers (*p* > 0.05). There was no statistically significant difference in the occurrence of menopause between the groups (*p* > 0.05). There was also no statistically significant difference in the occurrence of co-morbid diseases across the groups (*p* > 0.05).

There was a statistically significant difference between anti-oestrogen medication usage status according to group, however (*p* < 0.05). Participants in the breast cancer group were found to have a significantly higher rate of anti-oestrogen medication usage compared to participants in the control group. There was no significant difference in the usage of insulin between the groups (*p* > 0.05). However, there was a statistically significant difference in steroid usage across the groups (*p* < 0.05). Participants in the breast cancer group were found to have a significantly higher rate of steroid usage compared to those in the control group. Protection status did not exhibit a statistically significant difference between the groups, (*p* > 0.05), and smoking status did not exhibit a statistically significant difference between the groups either (*p* > 0.05) (Table 1).

Upon examination of the different HPV types, no statistically significant difference was seen in the number of types observed across the groups (*p* > 0.05). There was a statistically significant difference between the HPV type 16 incidence rates according to group (*p* < 0.05). The incidence of HPV type 16 in the control group was higher than in the breast cancer group. The incidence rates of HPV types 18, 31, 33, 35, 36, 39, 45, 52, 56, 58, 59, 66, and 68 did not show a statistically significant difference according to group (*p* > 0.05). There was a statistically significant difference between the incidence rates of HPV type “Other” according to group (*p* < 0.05). The incidences of HPV types 31, 52, 58, and “Other” in the breast cancer group were found to be higher than in the control group (Table 2).

There was no statistically significant difference between the smear results according to group (*p* > 0.05), between the colposcopy results according to group (*p* > 0.05), and between the ECC results according to group (*p* > 0.05) (Table 3).

## 5. Discussion

All cases examined in our study, which included both breast cancer and healthy cases, tested positive for HPV. Our objective was to determine whether tamoxifen use led to an increase in histopathological lesions on the cervix uteri, particularly in breast cancer patients who were treated with tamoxifen after surgery and adjuvant treatment. Based on the overall results of our study, there was no statistically significant difference found between the two groups in terms of smear cytology, colposcopic biopsy, and endocervical sample results. Overall, an analysis of every HPV-positive case revealed that the use of tamoxifen does not result in an increase in atypical changes in cervical lesions.

Based on the overall results of our study, there was no significant statistical difference observed between the group of individuals with breast cancer who used tamoxifen and the control group who underwent regular cytological screening in terms of age, gravida, parity, abortion rates, menopause status, co-morbidity rates, related insulin and steroid usage rates, contraceptive method usage rates, and smoking rates. All patients in our study tested positive for HPV following screening. The most common types of HPV were types 16 and 18, a finding that aligns with findings from previous studies. The absence of any difference between the groups in terms of demographic and clinical data is a significant finding in our study in terms of allowing for more distinct results regarding the impact of anti-oestrogen therapy as a chemo-preventive treatment for breast cancer on cervix uteri lesions in both groups examined.

Among women, breast cancer is the most prevalent form of cancer and the leading cause of cancer-related mortality. Surgery, chemotherapy, and radiotherapy to treat the disease are employed either individually or in combination. The course of treatment includes the administration of adjuvant chemotherapy and/or radiotherapy, in addition to the use of smart pharmacological therapies. This is followed by the use of selective oestrogen receptor agonists and aromatase inhibitor treatments as anti-oestrogen drugs used for chemoprevention, particularly to safeguard against the recurrence of lesions [1,2,3]. Tamoxifen is a commonly used drug for the treatment of all stages of breast cancer due to its high tolerability and limited adverse effects. Despite evidence suggesting a rise in pathologies affecting the female genital system during tamoxifen treatment, it continues to be employed as a prospective chemo-preventive agent, particularly among women who are at increased risk of breast cancer recurrence, owing to its favourable impact on both disease-free survival and overall survival from breast cancer, notwithstanding its potential adverse effects.

Tamoxifen is believed to have an impact on oestrogen receptors in cervicovaginal tissues. The results of the present study demonstrate the existence of oestrogenic receptors in both cervical squamous and columnar cells of both premenopausal and postmenopausal women [19]. Furthermore, tamoxifen is believed to diminish the impact of lymphocytic cells on tumour cells by suppressing the immune system. These immune-suppressive effects in the tumour microenvironment may lead to an elevation in the number of atypical cells detected during cervical cytological scans [22]. There is a need to examine the effects of tamoxifen and human papillomavirus (HPV) on abnormal cytologic changes. The objective in this case is to determine the efficacy of HPV in breast cancer patients treated with tamoxifen and to determine whether the atypical changes seen are caused by HPV or the impact of tamoxifen [19,22,23].

The authors of a study examining breast cancer cases, comparing those who used tamoxifen and those who did not, found that 61% of the cases exhibited atypical benign squamous cellular changes. Furthermore, the group taking tamoxifen showed a significantly higher occurrence of these atypical changes. Half of the detected abnormal changes were classified as ASC-US; the remaining cases, in comparison, did not show any signs of dysplastic changes. Furthermore, when a follow-up examination was performed, it was determined that none of the cases exhibited any malignant or premalignant changes. Furthermore, participants in the group that used tamoxifen showed no elevation in the incidence of bleeding or inflammation [16].

During the follow-up period, it was noted that postmenopausal breast cancer patients who were treated with tamoxifen experienced an increase in the maturation of squamous cells in cervicovaginal smears and an increase in the proliferation of the cervicovaginal epithelium. However, there was no observed increase in the incidence of cervical cancer in the cervix uteri compared to the control groups [18,23]. Nevertheless, the authors of another study also documented a rise in the frequency of isolated metastatic cervical uteri cancer, particularly among individuals with breast cancer who were using tamoxifen [24].

While tamoxifen functions as an oestrogen agonist in the uterus, it can be considered protective against cervical neoplasia. However, low-grade cervical dysplasia changes are rare in the general population, except in young individuals who undergo regular Pap smears. Although long-term use of tamoxifen in breast cancer patients actually increases the risk of endometrial and uterine stromal cancer, it has been stated in the literature that it does not lead to an increase in dysplastic changes in cervical cytology that could progress to neoplasia [16,25,26,27]. Indeed, the results of a phase II study indicated that the administration of tamoxifen had a beneficial therapeutic impact on the recurrence of non-squamous cell carcinomas in the cervix uteri [28].

It has been observed that a significant proportion of individuals who use aromatase inhibitors also use tamoxifen. In light of the fact that tamoxifen exhibits a neutral and moderately efficacious effect in mitigating the risk of cervical neoplasia, it is evident that aromatase inhibitors are more beneficial in reducing the risk of cervical neoplasia occurrence. Additionally, aromatase inhibitors are more effective than tamoxifen in preventing the recurrence of breast cancer, particularly in cases where the cancer is positive for oestrogen and/or progesterone receptors [29,30].

Based on experimental studies performed on HPV E6/E7 transgenic mice, it has been demonstrated that oestrogen and oestrogen receptor alpha are essential for the entire development process of cervical uteri carcinogenesis [31,32]. Furthermore, in another experimental model, it was shown that cervical neoplasias were absent in models where 17β-oestradiol was not supplied at normal levels and the oestrogen receptor gene was negative [33]. Furthermore, in another study, it was noted that cervical neoplasias experienced regression when a selective oestrogen receptor modulator was administered [34]. As a result, oestrogen and oestrogen receptor alpha are known to have a significant impact on the development of cervix uteri neoplasias. However, due to a scarcity of sufficient and reliable human studies, there is a lack of evidence pertaining to the role of oestrogen, oestrogen receptors, and selective oestrogen receptor modulator therapy in the development of cervical cancer [28,35,36].

Upon examination of the cytological and histopathological results of our HPV-positive control and breast cancer cases, it is evident that high-grade atypical lesions exhibited similarities in both the control group and the tamoxifen-use group. Furthermore, there was no significant statistical difference found between these two groups. Following cytological and colposcopy analyses, the primary objective of our study was to identify atypical changes in the epithelium of the cervical uterus induced by tamoxifen and chemo-radiotherapy methods used as adjuvant chemoprevention treatments for breast cancer patients who are HPV positive. Particularly in identifying cervical epithelial changes in their earliest stages, cervical cytological screening tests should be implemented similarly to those utilised in the general population.

In recent years, it has been stated that targeted therapies, especially in triple-negative breast cancer cases with a high risk of recurrence and metastasis, have positive effects on therapeutic efficacy and survival through chemotherapy, anti-angiogenesis, and immunotherapy [37].

The limitations of our study are the fact that it was retrospective in nature, the number of sexual partners of the participants was not included in our data, and the number of cases was low in both groups. However, the strengths of our study are that we avoided bias, there were cases using tamoxifen regularly for long-term prophylactic treatment, the participants’ cytological screenings were conducted regularly at a single centre for a long period of time, the study participants consisted of oestrogen and/or progesterone receptor-positive cases with no signs of recurrence in terms of breast cancer, the participants were followed up spontaneously, all cases were HPV positive, and the participants consisted of only cases using tamoxifen and included histopathological results. Due to the limited sample size in our study, we recommend performing further investigations in the future with a larger sample of groups by utilising innovative methodologies.

## 6. Conclusions

In light of the results presented herein, it appears that the use of tamoxifen in breast cancer patients with HPV-positive cytological screenings did not lead to an increase in abnormal histopathological lesions in the cervix uteri in the patients examined. Patients with breast cancer receiving follow-up and adjuvant chemoprevention therapy should be instructed to undertake annual gynaecological examinations and Pap smear testing.

## Figures and Tables

**Table 1:** Evaluation of clinical features according to group.

		Control Cases	Breast Cancer Cases	*p*-Value
Age (mean ± SD)		47.49 ± 7.95	46.73 ± 7.54	^1^ 0.530
Gravida (min–max/median)		0–8/2	0–7/2	^2^ 0.543
Parity (min–max/median)		0–6/2	0–5/1.5	^2^ 0.325
Abortion (min–max/median)		0–4/0	0–2/0	^2^ 0.830
D/C (min–max/median)		0–5/0	0–2/0	^2^ 0.789
Menopause (*n*; %)	Yes	97; 42.2	22; 42.3	^3^ 0.986
	No	133; 57.8	30; 57.7	
Co-morbidity (*n*; %)	Yes	90; 39.1	27; 51.9	^3^ 0.091
	No	140; 60.9	25; 48.1	
Anti-oestrogen (*n*; %)	Uses	0; 0	51; 98.1	^4^ 0.001 **
	Not Using	230; 100	1; 1.9	
Insulin (*n*; %)	Uses	27; 11.7	6; 11.5	^4^ 1.000
	Not Using	203; 88.3	46; 88.5	
Steroid (*n*; %)	Uses	23; 10.0	11; 21.2	^4^ 0.046 **
	Not Using	207; 90	41; 78.8	
Contraception (*n*; %)	Yes	68; 29.1	13; 25	^4^ 0.626
	No	162; 70.4	39; 75	
Smoking (*n*; %)	Yes	96; 41.7	21; 4 0.4	^3^ 0.858
	No	134; 58.3	31; 59.6	

^1^ Student’s *t*-test. ^2^ Mann–Whitney U test. ^3^ Ki–Kare test. ^4^ Yates continuity test. D/C: dilate and curettage. ** *p* < 0.05.

**Table 2:** Evaluation of HPV types by group.

		Control Cases	Breast Cancer Cases	*p*-Value
HPV Type Number (*n*; %)	Single	75; 32.6	14; 26.9	^4^ 0.528
	Multiple	155; 67.4	38; 73.1	
HPV Type (*n*; %)	Type 16	135; 58.7	22; 42.3	^3^ 0.032 *
	Type 18	78; 33.9	16; 30.8	^4^ 0.786
	Type 31	30; 13	9; 17.3	^4^ 0.561
	Type 33	29; 12.6	6; 11.5	^4^ 1.000
	Type 35	11; 4.8	1; 1.9	^5^ 0.702
	Type 39	6; 2.6	0; 0	^5^ 0.597
	Type 45	27; 11.7	6; 11.5	^4^ 1.000
	Type 52	21; 9.1	8; 15.4	^4^ 0.277
	Type 56	12; 5.2	1; 1.9	^5^ 0.474
	Type 58	12; 5.2	3; 5.8	^5^ 0.744
	Type 59	6; 2.6	0; 0	^5^ 0.597
	Type 66	2; 0.9	0; 0	^5^ 1.000
	Type 68	2; 0.9	0; 0	^5^ 1.000
	Others	21; 9.1	12; 23.1	^4^ 0.010 *

^3^ Ki–Kare test. ^4^ Yates continuity correction test. ^5^ Fisher’s exact test. * *p* < 0.05. HPV: human papillomavirus.

**Table 3:** Evaluation of the distribution of smear cytology, colposcopy, and ECC results according to group.

		Control Cases	Breast Cancer Cases	*p*-Value
Smear cytology	NILM	46; 20	17; 32.7	0.271
	ASC-US	43; 18.7	6; 11.5	
	LGSIL	72; 31.3	16; 30.8	
	HGSIL	41; 17.8	8; 15.4	
	ASC-H	16; 7	5; 9.6	
	AGC-NOS	11; 4.8	0; 0	
	AIS	1; 0.4	0; 0	
Colposcopy	NILM	2; 0.9	1; 1.9	0.913
	Chronic cervicitis	38; 16.5	9; 17.3	
	LGSIL	114; 49.6	26; 50	
	HGSIL	76; 33	16; 30.8	
ECC	Negative	130; 56.5	27; 51.9	0.877
	Chronic cervicitis	27; 11.7	8; 15.4	
	HGSIL	28; 12.2	6; 11.5	
	LGSIL	45; 19.6	11; 21.2	

The chi-squared test was used. NILM: negative for intraepithelial lesion or malignancy. ASC-US: atypical squamous cells of undetermined significance. LGSIL: low-grade squamous intraepithelial lesion. ASC-H: atypical squamous cells, cannot exclude high-grade squamous intraepithelial lesions. HGSIL: high-grade squamous intraepithelial lesion. AGC-NOS: atypical glandular cells not otherwise specified. AIS: adenocarcinoma in situ.

## Data Availability

The datasets used and/or analysed during the present study are available from the corresponding author upon reasonable request.
